# Implementation Science for HIV Prevention and Treatment in Indigenous Communities: a Systematic Review and Commentary

**DOI:** 10.1007/s11904-024-00706-z

**Published:** 2024-08-09

**Authors:** Christopher G. Kemp, Abagail J. Edwards, Lauren White, Gauri Kore, Pamela Jumper Thurman, Tommi Gaines, Paula Toko King, Marama Cole, E. Roberto Orellana

**Affiliations:** 1 Center for Indigenous Health, Department of International Health, Johns Hopkins Bloomberg School of Public Health, Baltimore, MD USA; 2 Joint Program for Social Work and Psychology, University of Michigan, Ann Arbor, MI USA; 3 Department of Epidemiology, Johns Hopkins Bloomberg School of Public Health, Baltimore, MD USA; 4 Department of Ethnic Studies, Colorado State University, Fort Collins, CO USA; 5 Department of Medicine, University of California San Diego, San Diego, CA USA; 6 Te Rōpū Rangahau Hauora a Eru Pōmare, Department of Public Health, University of Otago, Wellington, New Zealand; 7 Indigenous Wellness Research Institute, University of Washington, Seattle, WA USA

**Keywords:** HIV, Implementation science, Indigenous, Community-based participatory research

## Abstract

**Purpose of Review:**

We systematically reviewed implementation research conducted in Indigenous communities in the Americas and the Pacific that focused on improving delivery of HIV preventive or treatment services. We highlight strengths and opportunities in the literature and outline principles for Indigenous-led, HIV-related implementation science.

**Recent Findings:**

We identified 31 studies, revealing a consistent emphasis on cultural tailoring of services to Indigenous communities. Common barriers to implementation included stigma, geographic limitations, confidentiality concerns, language barriers, and mistrust. Community involvement in intervention development and delivery emerged as a key facilitator, and nearly half of the studies used community-based participatory research methods. While behavioral HIV prevention, especially among Indigenous youth, was a major focus, there was limited research on biomedical HIV prevention and treatment. No randomized implementation trials were identified.

**Summary:**

The findings underscore the importance of community engagement, the need for interventions developed within Indigenous communities rather than merely adapted, and the value of addressing the social determinants of implementation success. Aligned to these principles, an indigenized implementation science could enhance the acceptability and reach of critical HIV preventive and treatment services in Indigenous communities while also honoring their knowledge, wisdom, and strength.

**Supplementary Information:**

The online version contains supplementary material available at 10.1007/s11904-024-00706-z.

## Introduction

HIV remains a significant public health challenge in Indigenous communities across the Americas and the Pacific. Reported rates of HIV diagnosis are disproportionately high for Indigenous peoples in Australia, Canada, Aotearoa/New Zealand, and the United States (US) [1]. In the U.S., between 2017 and 2021, annual HIV diagnoses among American Indian/Alaska Native (AI/AN) people increased by 16% and diagnoses among Native Hawaiians and other Pacific Islanders increased 55%; diagnoses decreased for all other racial and ethnic groups [2]. AI/AN people also have the shortest survival time after diagnosis among racial and ethnic groups in the US, reflecting inequities in access to testing and treatment uptake [3]. HIV prevalence in Indigenous communities in Venezuela (Warao), Peru (Chayahuita), and Colombia (Wayuu women) has been estimated at 9.6%, 7.5%, and 7.0%, respectively, substantially higher than the general population average of 0.4% in the region [4, 5]. Papua New Guinea has the highest incidence and prevalence of HIV in the Pacific, and infection rates there are steadily increasing [6]. Multiple factors contribute to these inequities, including differential exposure to the social determinants of health, with Indigenous communities often facing higher rates of poverty and unemployment than non-Indigenous communities [7, 8]. Limited access to healthcare – often exacerbated by remote living conditions, a lack of culturally safe services, and chronic under-funding in violation of treaty agreements – further hinders effective HIV prevention and treatment [9, 10]. Furthermore, the effects of colonization, coloniality, racism, and discrimination, which have disrupted traditional social structures and introduced new vulnerabilities, play a significant role in the current HIV epidemic in these populations [8, 11]. Addressing these challenges requires a multifaceted approach that respects and integrates Indigenous knowledge, values, and systems [12].

As noted in the literature, inequities in the implementation of health interventions for different population groups contribute to differential health benefits [13]. Thus, implementation science, which focuses on understanding and addressing barriers to the effective adoption of evidence-based interventions, has the potential to help to bridge these gaps. As a relatively new, rapidly growing field using a range of interdisciplinary methods, implementation science is unique in its focus on the ‘how’ of health service delivery in the real world [14]. Core to the science are implementation strategies – deliberate approaches to facilitate intervention delivery, including training, financial incentives, and audit and feedback [15, 16]. Implementation science measures the outcomes of these strategies, focusing on concepts like acceptability, feasibility, fidelity, and sustainability [17]. Implementation science with a health equity focus could thus offer insights into appropriate strategies for implementing HIV prevention and treatment services in Indigenous communities, including the tailoring of culturally safe services. to align with local priorities, practices, and knowledges, thereby leveraging their known strengths [10].

Given the urgent need to improve HIV outcomes in Indigenous communities and the potential of implementation science to support this goal, we sought to understand the scope of current research in this area. Our objectives were to review implementation research conducted in and with Indigenous communities in the Americas and the Pacific that focused on improving delivery of HIV prevention or treatment services, with the intent of outlining principles for future Indigenous-led, HIV-related implementation science.

## Methods

### Search Strategy

We searched PubMed on June 29, 2023 to identify original peer-reviewed research in any language that 1) evaluated the implementation of HIV preventive or treatment interventions, 2) assessed at least one implementation outcome as specified by Proctor et al. (2011) or Glasgow et al. (1999) [17, 18], and 3) enrolled from a majority Indigenous population in North America, Central America, South America, or the Pacific (i.e., Australia, Aotearoa/New Zealand, Polynesia, Micronesia, and Melanesia). The full search strategy is presented in Additional File 1.

### Study Selection

During the title and abstract screening phase, all database results were uploaded into ASReview, an active learning tool designed to assist systematic review screening by automatically categorizing results by relevance [19]. Prior research has shown that ASReview's algorithm can identify 95% of the final selected publications within the initial 20% of the publications shown, significantly reducing the time required for screening while ensuring the quality and integrity of the results [20]. The first author (CK) manually reviewed all results using ASReview. Studies were included at the title and abstract screening phase if they appeared to be related to HIV/AIDS in Indigenous communities. We then used Covidence for full-text screening [21]. A mix of two authors (CK, AE, GK, or LW) independently screened all full-text articles and noted reasons for exclusion. Studies passed the full-text screening stage if they met all inclusion criteria. Discrepancies in eligibility assessments were resolved through discussion until consensus was reached.

### Data Abstraction

Two authors (CK and AE) independently piloted a structured abstraction form on Covidence. One of four authors (CK, AE, GK, or LW) then abstracted study, intervention, and implementation strategy characteristics for the remaining studies, while another author independently verified each abstraction, and then resolved any disagreement through discussion. We abstracted study settings, objectives, study design and methods, whether community-based participatory methods were used, whether any author-identified Indigenous research methods were used, study populations, HIV prevention or treatment interventions of focus, types of implementation strategies used [22] – including author-defined Indigenous implementation strategies, implementation outcomes reported [17, 18], HIV-related outcomes reported, and conclusions or lessons learned. Risk of bias was not assessed as no meta-analysis of effectiveness was conducted.

### Analysis

Percentages were calculated for all categorical variables; these were used to summarize study characteristics. Quantitative meta-analysis of study findings was not possible given the heterogeneity in research questions and outcomes.

## Results

**Fig. 1:**
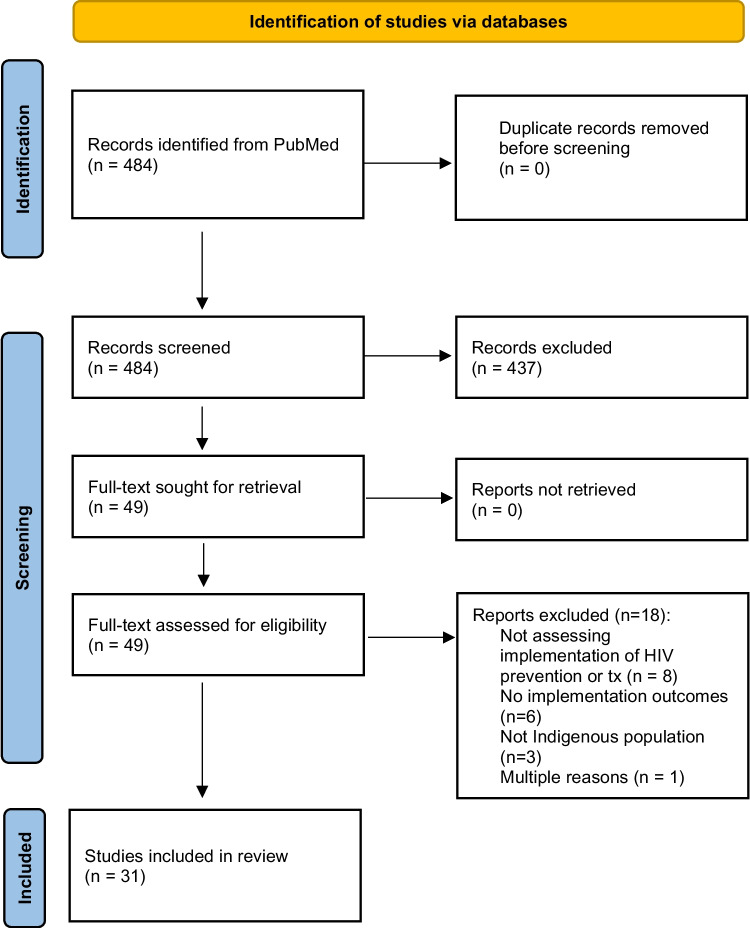
PRISMA 2020 flowchart of systematic review

Our search yielded 484 articles. We excluded 435 during title/abstract screening, leaving 49 for full-text review. Of these, eight were excluded for not assessing implementation of an HIV treatment or preventive intervention, six were excluded because they did not plan to measure or report an implementation outcome, three were excluded because they were not conducted in or with an Indigenous community, and one was excluded for multiple reasons (Fig. 1).

The final sample included 31 studies (Table 1) [23–53]. Table 2 provides descriptive statistics. The largest number (10, 32.3%) were conducted in Canada, followed by the United States (9, 29.0%) and Australia (4, 12.9%). Two (6.5%) were study protocols; the rest presented empirical data. A range of formative and evaluative study designs were adopted; cross-sectional qualitative or survey designs (9, 29.0%) and quasi-experimental designs, including pre-post without control designs (8, 25.8%), were the most used. Nearly half (15, 49.4%) of studies used key-informant interviews. Community-based participatory research methods were clearly specified in fifteen (48.4%) studies. Indigenous research methods used included gathering or talking circles and the Aboriginal ownership, control, access, and possession (OCAP) model [54]. Most studies evaluated implementation of behavioral HIV prevention programs (17, 54.8%), and twelve (38.7%) evaluated implementation of testing programs.

**Table 1:** Included studies, by year of publication and author (*n* = 31)

First author & citation	Year	Location	Study design	Indigenous method	Community	Interventions	Implementation strategies	Indigenous strategies	Implementation outcomes	Key findings
Crown [23]	1993	Canada	Case study	None	Inuit, Dene First Nations, Métis	BehP	EC	None	Ac; Ap	Respect for elders and obtaining appropriate support from chiefs and band councils promoted community ownership and receptivity to the campaign
Baldwin [24]	1996	United States	Retrospective process evaluation	None	American Indian/Alaska Native	BehP	TES	None	Ac; Ad; Ap	Pilot curriculum perceived as useful but should be expanded to be more reflective of traditional Native American approaches to health, more visual and action-oriented
Miller [25]	1998	Australia	Quasi-experimental	None	Aboriginal (Pitjantjatjara)	T	SC; TES; UEIS	None	R	Increase and sustainment of HIV testing rates after new guidelines
Aguilera [26]	2005	United States	Case study	None	Urban Native	BehP	DSI; EC; PIA; TES	None	Ac; R	Participants learned about Native American culture and felt more connected; drug refusal skills improved; most would be more involved in community activities
Mikhailovich [27]	2005	Australia	Quasi-experimental	None	Aboriginal	BehP	EC; TES	None	Ac; Ad; S	Program trained young Indigenous peer educators and developed and disseminated more than 2,600 sexual health education resources to young Indigenous people and their community. Arts-based strategies helped young people to remain engaged and enthusiastic
Bucharski [28]	2006	Canada	Cross-sectional	None	First Nations, Métis	T	ATC	None	Ac; Ap	Recommends Aboriginal determination of Aboriginal women in HIV policy and programming, harm reduction approach, and employing a tester who is sensitive to the multiple hardships and issues Aboriginal women
Andersson [29]	2008	Canada	Quasi-experimental	Talking circles; OCAP	Aboriginal	O	ATC; DSI; EC; TES; UEIS	Elders as stakeholders	Ac; Ad; Ap; Fe; R	Project will build capacity within communities to identify strategies related to resilience that can be incorporated into public health and clinical practice
Barlow [30]	2008	Canada	Cross-sectional	OCAP	Métis, Inuit	O	ATC; DSI; TES	None	Ac; Ad; Ap	Addictions and HIV must be treated together, reflecting a holistic worldview of Aboriginal people
Lowe [31]	2008	United States	Quasi-experimental	Cherokee self-reliance questionnaire	Cherokee	BehP		Talking circles	Fe	HCWs could use approaches like the Talking Circle when planning and implementing prevention programs for Native American youth
Craig Rushing [32]	2012	United States	Formative strategy design	Tribal coalition	Northwest Tribes	BehP	ATC	None	Ac; Ap; Fe	Partners discussed the effectiveness of various technology-based interventions, and design features that have been shown to maximize behavioral impacts
Newman [33]	2012	Canada	Cross-sectional	None	Urban Aboriginal	BioP		None	Ac; Ap	Vaccine uptake motivated by community survival. Negative HIV vaccine perceptions, mistrust of government and healthcare institutions, perceived conflict between western and traditional medicine, sexual prejudice, AIDS stigma, and vaccine cost may limit vaccine acceptability. Suggest building on cultural strengths and acknowledging history of mistrust and social exclusion
Tu [34]	2013	Canada	Cohort	None	Aboriginal	T; Tx	ATC; CI; SC; TES; UEIS	None	Ad; Fi; R	Chronic care model encourages adoption of clinical practice guidelines, empowers care providers to proactively identify patients in need of intervention, and encourages patients to be more active in their self-care
Benzaken [35]	2014	Brazil	Quasi-experimental	None	Amazon Indigenous	T	TES	None	Fe	Few HCWs reported difficulties in performing POC tests
Ribeiro [36]	2015	Brazil	Cross-sectional	None	Amazon Indigenous	T	CI; TES	None	Ad	High rate of acceptance of HBCT
Ruffinen [37]	2015	Brazil	Prospective process evaluation	None	Amazon Indigenous	T	ATC; UEIS	None	Ac; Ad; Ap; Fe; P; R; S	Results will inform strategies to improve feasibility, viability, and sustainability of introducing HIV and syphilis POC testing in the Amazon, including addressing the preparation phase at the coordination and training levels
Craig Rushing [38]	2016	United States	Formative intervention design	None	American Indian/Alaska Native	BehP	ATC; TES; UEIS	None	Ac; Ap; Fe	Youth, parents, and tribal health educators rated video as culturally appropriate and felt information could be trusted. Staff offered suggestions to improve usability and implementation
Ansari [39]	2017	Indonesia (West Papua)	Cohort	None	Papuan	BioP	CI; EC	None	Ac; Fe	Majority of clients were satisfied and would recommend non-surgical circumcision to family and friends
Shegog [40]	2017	United States	Case study	Culturally sensitive adaptation framework	American Indian/Alaska Native	BehP	ATC	None	Ac; Ad; Ap; Fe	Youth rated lessons as enjoyable and easy to use. Stakeholders described the language as empowering, culturally appropriate, and representative of the student perspective
Lee [41]	2018	United States	Quasi-experimental	None	Urban Native	BehP	ATC	None	Ac; Ap	Adapted BART curriculum was culturally responsive and acceptable to the Native American youth participants in an urban-based geographical setting
Palma-Pinedo [42]	2018	Peru	Cross-sectional	None	Peruvian Indigenous	T	ATC; UEIS	None	Ac; Ad; Ap; C; Fe; R	Geographic, sociocultural, and health system barriers identified, including reagent shortages and limited budget
Treloar [43]	2018	Australia	Quasi-experimental	None	Aboriginal and Torres Strait Islander	T; BehP	ATC; DSI; UFS; O	None	Ac; Ad; Ap; Fe; P; R	Significant engagement by Aboriginal people, high acceptability, requiring modest incentives
Larcombe [44]	2019	Canada	Quasi-experimental	None	Dene First Nations	T; BehP; Tx; O	PIA; TES; UEIS	Community readiness assessment	Ac; Ad; Ap	Training adaptations to increase interaction and discussion Priorities identified for adult education and youth involvement in programs and planning
Jongbloed [45]j	2020	Canada	Cohort	None	First Nations, Inuit, Métis	T; BehP; BioP; Tx, O	EC; PIA	None	Ac; Ap	High acceptability of mobile phones for health
Worthington [46]	2020	Canada	Retrospective process evaluation	None	First Nations	BehP	ATC; DSI; TES; UEIS; UFS	None	Ac; Ap; Fe; S	Key lessons include involving target communities in program development; prioritizing community partnerships; building relationships; local relevancy and appropriateness; assessing community readiness; and program flexibility & adaptability
Kaufman [47]	2021	United States	Cross-sectional	None	American Indian/Alaska Native	BehP		None	Ac; Ap; Fe; O	Positive support for RESPECT, especially related to observability, complexity, and compatibility
Ubrihien [48]	2021	Australia	Cross-sectional	None	Aboriginal and Torres Strait Islander	Tx		None	Ac; Ap; Fe	Will provide policy makers with practical measures to improve cultural appropriateness and clinical care of young Aboriginal people
Gabster [49]	2022	Panama	Cross-sectional	None	Guaymí, Ngäbe, Buglé	Tx		None	Ad; Ap; Fe	ART shortages impact adherence. Uncooperative interaction between the traditional and Western health systems may be detrimental
Landy [50]	2022	Canada	Prospective process evaluation	Gathering circles	Métis	T	ATC; EC	None	Ac; Ap; Fe	DBST is highly acceptable among community members
Markham [51]	2022	United States	Formative strategy design	None	American Indian/Alaska Native	BehP	ATC; DSI; PIA; TES; UEIS	None	Ac; Ad; Ap; Fe	Healthy Native Youth Implementation Toolbox supports Native practitioners to adopt, implement, and maintain a culturally relevant, age-appropriate sexual health EBP
Sianturi [52]	2022	Indonesia (West Papua)	Cross-sectional	None	Papuan	T; BioP; Tx	ATC; CI	None	Ac; Ap; Fe	Highlighted need for programs to be sensitive toward culture
Nogueira [53]	2023	Guatemala	Prospective process evaluation	None	Mayan	BehP; BioP	ATC	None	Ac; Ap; Fe	Adaptation widely accepted and culturally appropriate and relevant among Mayan *comadronas*

Abbreviations:* T* Testing; *BehP* Behavioral prevention; *BioP* Biomedical prevention; *Tx* HIV treatment; *O* Other; *ATC* Adapt and tailor to context; *CI* Change infrastructure; *DSI*, Develop stakeholder interrelationships; *EC* Engage consumers; *PIA* Provide interactive assistance; *SC* Support clinicians; *TEA* Train and educate stakeholders; *UEIS* Use evaluative and iterative strategies; *UFS* Utilize financial strategies; *Ac* Acceptability; *Ap* Appropriateness; *Ad* Adoption; *C* Costs; *Fe* Feasibility; *Fi* Fidelity; *P* Penetration; *S* Sustainability

**Table 2:** Study-level descriptive statistics (*n* = 31)

	*N* (%)
Year (median [IQR])	2016 [2008, 2020]
Location	
Australia	4 (12.9)
Brazil	3 (9.7)
Canada	10 (32.3)
Guatemala	1 (3.2)
Indonesia (West Papua)	2 (6.5)
Panama	1 (3.2)
Peru	1 (3.2)
United States	9 (29.0)
Study protocol	2 (6.5)
Study design	
Case study	3 (9.7)
Cohort	3 (9.7)
Cross-sectional	9 (29.0)
Formative intervention design	1 (3.2)
Formative strategy design	2 (6.5)
Prospective process evaluation	3 (9.7)
Quasi-experimental	8 (25.8)
Retrospective process evaluation	2 (6.5)
Data collection tools*	
Focus group discussions	12 (38.7)
Key informant interviews	15 (48.4)
Surveys	9 (29.0)
Observation	1 (3.2)
Advisory groups	1 (3.2)
Other	2 (6.5)
Community-based participatory research	
No	15 (48.4)
Unclear	1 (3.2)
Yes	15 (48.4)
Indigenous research methods*	
Aboriginal ownership, control, access, and possession model	2 (6.5)
Cherokee self-reliance questionnaire	1 (3.2)
Cultural sensitivity adaptation framework	1 (3.2)
Gathering or talking circles	2 (6.5)
Tribal coalition	1 (3.2)
HIV preventive or treatment intervention of focus*	
Testing	12 (38.7)
Behavioral prevention	17 (54.8)
Biomedical prevention	4 (12.9)
HIV treatment	6 (19.4)
Other	5 (16.1)
Implementation strategies used*	
Adapt and tailor to context	16 (51.6)
Change infrastructure	4 (12.9)
Develop stakeholder interrelationships	6 (19.4)
Engage consumers	7 (22.6)
Provide interactive assistance	4 (12.9)
Support clinicians	2 (6.5)
Training and educate stakeholders	13 (41.9)
Use evaluative and iterative strategies	9 (29.0)
Utilize financial strategies	2 (6.5)
Other	1 (3.2)
Indigenous implementation strategies	
Community readiness model	1 (3.2)
Elders as stakeholders	1 (3.2)
Talking circles	1 (3.2)
Implementation outcomes planned or reported*	
Acceptability	25 (80.6)
Adoption	13 (41.9)
Appropriateness	23 (74.2)
Cost	1 (3.2)
Feasibility	18 (58.1)
Fidelity	1 (3.2)
Penetration	2 (6.5)
Reach	7 (22.6)
Sustainability	2 (6.5)
Other	1 (3.2)
HIV-related outcomes planned or reported*	
HIV/sexual health-related knowledge/awareness	8 (25.8)
Testing	8 (25.8)
Knowledge of status	2 (6.5)
Linkage to prevention or treatment	1 (3.2)
Treatment or prevention initiation	0 (0)
Treatment or prevention adherence	2 (6.5)
Retention in care	1 (3.2)
Viral suppression	1 (3.2)
Other	4 (12.9)

* ≥ 1 response per study possible

Studies described the use of a range of implementation strategies to support program implementation. The most common strategies included adaptation and tailoring of interventions (16, 51.6%) for implementation in Indigenous communities. Strategies to train and educate stakeholders were also common (13, 41.9%). Indigenous implementation strategies included the community readiness model [55], the intentional involvement of elders as stakeholders, and the use of talking circles [56]. Acceptability was the most common implementation outcome reported (25, 80.6%), followed by appropriateness (23, 74.2%), feasibility (18, 58.1%), and adoption (13, 41.9%). Sustainability (2, 67.5%), cost (1, 3.2%), and fidelity (1, 3.2%) were rarely assessed. Common HIV-related outcomes included knowledge or awareness of HIV and sexual health (8, 25.8%) and testing (8, 25.8%). Later-stage HIV-related outcomes (e.g., viral suppression) were rarely assessed.

We organize our summary of HIV-related implementation research in Indigenous communities by the prevention or treatment interventions of focus in each study. Studies evaluating implementation of multiple interventions are categorized by the most upstream intervention (i.e., testing, then behavioral prevention, then biomedical prevention, then treatment, then other interventions).

### HIV Testing

Three studies explored the perspectives of different Indigenous communities on HIV testing. Bucharski et al. (2006) conducted a study with Canadian Aboriginal women, noting several barriers to testing uptake but also identifying guiding principles for culturally appropriate testing programs [28]. Palma-Pinedo and Reyes-Vega (2018) conducted a similar study in the Peruvian Amazon and found barriers including geographic limitations, sociocultural challenges, confidentiality concerns, language barriers, mistrust of the screening process, and limited healthcare resources [42]. They also emphasized the need for culturally sensitive and differentiated care. Finally, Sianturi et al. (2022) conducted a study in Indonesia to understand the reasons for the lack of acceptance of HIV programs among Indigenous Papuans. They argued for community-based, multi-sectoral, culturally sensitive approaches to educating and building awareness around HIV [52].

Five studies assessed the acceptability, feasibility, and uptake of specific testing approaches. Miller and Torzillo (1998) evaluated the uptake of HIV testing in remote Aboriginal communities in Australia, crediting the high uptake among high-risk groups to the confidentiality that was maintained and to the use of community-wide education [25]. Three studies were related and conducted with Indigenous communities in the Brazilian Amazon. Benzaken et al. (2014) demonstrated the feasibility of dried tube specimens (DTS) for external quality assurance of point-of-care syphilis and HIV testing [35]. Ruffinen et al. (2015) assessed the implementation of a point-of-care screening program for syphilis and HIV in these communities, describing the context for the introduction of the testing, evaluating the performance of the healthcare system, and describing barriers to and facilitators of implementation success. Their results formed the basis for the design of strategies to improve the feasibility, viability, and sustainability of introducing point-of-care syphilis and HIV testing on a larger scale in the Amazon [37]. Finally, Ribeiro et al. (2015) demonstrated the acceptability of home-based, voluntary counselling and testing (HBCT) for HIV and syphilis, estimated the prevalence of both conditions, and assessed the performance of point-of-care testing by healthcare staff using DTS. They noted high acceptance of HBCT by community members [36]. Separately, Landy et al. (2022) explored the acceptability of dried blood spot testing (DBST) for HIV, STIs, and blood-borne infections among Métis people in Alberta, Canada. They used a mixed-methods approach, including gathering circles, and found that DBST was highly acceptable to Métis community members and could be part of a culturally grounded, Métis-specific epidemic response [50].

Two studies evaluated more comprehensive testing related intervention packages. Treloar et al. (2018) assessed the acceptability of the Deadly Liver Mob program, which was aimed at engaging Aboriginal Australians in hepatitis C and sexual health education, screening, and care, including educational sessions about HIV and referral to a sexual health service for HIV assessment and screening. They found that the program was acceptable to staff and clients and was effective in increasing the proportion of Aboriginal clients attending health education and screening services [43]. Tu et al. (2013) discussed the implementation of the chronic care model (CCM) to improve HIV care in a predominantly Indigenous population in Canada. The CCM includes enhancing clinical teamwork, promoting evidence-based clinical recommendations, empowering patients to manage their own care, and creating a framework for population-based quality improvement initiatives. The authors found that the CCM led to improvements in HIV implementation outcomes, including increased rates of testing, treatment uptake, and effectiveness outcomes, such as viral suppression [34].

### Behavioral Prevention

Behavioral HIV prevention with Indigenous youth was a major focus; most of these studies used community-based, culture-forward approaches, and authors emphasized the importance of community involvement and cultural relevance in successful program adoption, implementation, and maintenance. Baldwin et al. (1996) documented the collaborative development and implementation of culturally sensitive HIV/AIDS and substance abuse prevention curricula for Native American youth, demonstrating the adaptability of multi-component preventive intervention curricula for Native American communities when combined with formative research activities and community input [24]. Aguilera and Plasencia (2005) described programs hosted by the Native American Health Center's Youth Services that incorporate traditional cultural activities and empowerment to reduce risk. The authors emphasized the importance of community healing, healthy traditions, and family involvement [26]. Mikhailovich and Arabena (2005) reported on the Indigenous Peer Education Project (IPEP), which trained young Indigenous Australians to become sexual health peer educators, finding positive effects on participants' knowledge and skills in sexual health education [27]. Lowe (2008) used a measure of Cherokee self-reliance and conducted a feasibility study using talking circles – a traditional coming-together approach – to deliver HIV/AIDS and HCV prevention material to Native American adolescents [31].

Four of these studies were related. Craig Rushing and Stephens (2012) first described the work of Project Red Talon – a STD/HIV prevention project with the Northwest Portland Area Indian Health Board Tribal Epidemiology Center – and their use of community-based participatory research methods to review existing technology-based interventions and generate recommendations for designing culturally appropriate media-based interventions for Native youth [32]. Craig Rushing and Gardner (2016) then described the adaptation process for a video-based HIV/STI intervention (Native VOICES) using the ADAPT-ITT model, including the development of a culturally tailored intervention toolkit [38, 57]. Shegog et al. (2017) also described the adaptation process of the Native It's Your Game curriculum, which included a needs assessment and the development of a web-based curriculum incorporating Native culture and language, all informed by cultural sensitivity adaptation frameworks and principles [40, 58]. Finally, Markham et al. (2022) detailed the development of the Healthy Native Youth Implementation Toolbox, which is a decision support system for implementing culturally-relevant sexual health education programs, adapted from the iCHAMPSS (CHoosing And Maintaining Effective Programs for Sex Education in Schools) toolkit using the process of implementation mapping [51, 59].

As part of a separate effort, Lee et al. (2018) described the adaptation of an HIV prevention intervention (Becoming a Responsible Teen, BART) for Native American adolescents. The authors received input from an advisory board, modified the intervention to be more consistent with Native American culture, and conducted a pilot study, finding that the adapted intervention was highly acceptable [41]. Kaufman et al. (2021) conducted a national survey of stakeholders involved in sexual health programs for Native American youth and sought to understand the factors that might facilitate or hinder their use of a particular evidence-based risk reduction intervention. They found that perceived trialability, compatibility, and observability all influenced the likelihood of intervention uptake [47].

In the oldest study in our sample, Crown et al. (1993) documented the challenges faced by Canada’s Northwest Territories in implementing HIV prevention strategies, including language barriers, cultural taboos, and confidentiality concerns, noting that programs were facilitated by the involvement of community members and the efforts of Community Health Representatives [23]. Worthington et al. (2020) also conducted a qualitative study on rural and remote regions community-based HIV/AIDS prevention interventions in Canada, highlighting the importance of involving communities in program development, building relationships and partnerships, assessing community readiness, program flexibility, and addressing stigma [46].

In the most recent study in our sample, Nogueira et al. (2023) aimed to culturally adapt an evidence-based HIV intervention for traditional birth attendants (*comadronas*) in rural Guatemala. The study found that the adapted intervention was acceptable, suitable, and feasible for the *comadronas*, and increased their confidence in HIV prevention [53].

### Biomedical Prevention

Two studies focused only on biomedical HIV prevention. Newman et al. (2012) examined the acceptability of a vaccine for HIV among sexually diverse Aboriginal peoples in Canada, identifying barriers to acceptance including mistrust, concerns about safety and efficacy, stigma, and cost. They emphasize the need for culturally appropriate dissemination approaches, including community engagement and working with local leaders [33]. Ansari et al. (2017) conducted a study in Papua, Indonesia to assess the acceptability and feasibility of voluntary medical male circumcision (VMMC), finding initially that demand was weak due to lack of prior socialization and concerns about safety and religious appropriateness [39].

### Treatment

Two studies focused on HIV treatment. Ubrihien et al. (2021) described a study protocol aiming to improve STI treatment outcomes for Aboriginal young Australians by addressing barriers to accessing sexual health services [48]. Gabster et al. (2022) similarly used interviews to assess the barriers and facilitators to treatment adherence and retention in HIV care among the Indigenous population in the Ngäbe-Buglé Comarca, Panama. Identified barriers included psychological health, family and community support or discrimination, and difficulties in accessing ART care due to travel costs, ART shortages, and challenges in navigating between Western and Traditional medical systems. One of their recommendations was to foster formal collaboration between Western and Traditional providers [49].

### Other

Four studies were concerned with HIV services generally. Two were from Australia. Andersson et al. (2008) outlined the protocol for the Aboriginal Community Resilience to AIDS (ARCA) research project, which aimed to investigate the role of resilience in the health and well-being of Canadian Aboriginal youth in relation to STIs and blood-borne viruses, using both talking circles and the OCAP model [29]. Barlow et al. (2008) further explored the issue of culturally competent service provision for Aboriginal people living with HIV/AIDS in Canada, again using the OCAP model. They also highlighted the importance of treating addictions and HIV/AIDS together [30].

Two studies in Canada related to identifying community needs and resources. Larcombe et al. (2019) described a pilot project by the Dene First Nations community in northern Manitoba, using both the community readiness model and OCAP model to develop culturally appropriate HIV-related interventions and programs [44]. Jongbloed et al. (2020) conducted a study of mobile phone ownership and usage among young Indigenous people in British Columbia who have used drugs with the goal of understanding challenges and potential solutions for engaging them in mobile health programs related to HIV and other conditions [45].

## Discussion

We identified 31 implementation research studies related to HIV prevention or treatment services in Indigenous communities in the Americas and the Pacific. Studies consistently emphasized the value of culturally safe services that are appropriately tailored to meet the needs and work in tandem with the strengths of Indigenous communities. Geographic limitations, confidentiality concerns, language barriers, mistrust, and insufficient healthcare resources were commonly identified barriers to implementation. Community involvement in intervention development, adaptation, and delivery was consistently noted as a key implementation facilitator, and around half of the studies used community-based participatory research methods. The largest number of studies were focused on behavioral HIV prevention, particularly among Indigenous youth, again using community-based, culture-forward approaches. Relatively few studies were focused on biomedical HIV prevention, with none evaluating programs seeking to improve access to or uptake of Pre-Exposure Prophylaxis (PrEP), and few related to HIV treatment. No randomized implementation trials were identified.

Our results highlight the growing role of implementation research in supporting HIV services for Indigenous communities. Studies used a diverse range of implementation research methods and strategies, uniquely incorporating several Indigenous approaches, including talking circles, for both data collection and intervention delivery. The absence of randomized trials in our sample is consistent with the observation that such trials may be considered culturally inappropriate in some Indigenous communities [60]. Studies predominantly focused on early-stage implementation outcomes such as patient- and provider-level acceptability and feasibility, finding that confidentiality, community education, and cultural adaptation improved intervention user perceptions of satisfaction and fit. However, in alignment with most other implementation research, few studies measured later-stage implementation outcomes like fidelity, cost, or sustainability [61–64]. Maintaining fidelity is vital to ensuring interventions work as intended [65]. Because a substantial number of HIV implementation studies include community-engaged methodologies, added attention to fidelity may inform our understanding of how implementation practitioners can hold the tension between community implementation and fidelity in Indigenous communities (e.g., Fidelity-Adaptation Dilemma [66]). Demonstrating cost, cost-effectiveness, and sustainability is crucial for justifying expansion, especially with constrained resources [67]. For example, healthcare for Indigenous communities in North America is drastically under-funded: the per capita Indian Health Service (IHS) funding allocation is approximately one third of US per person health care spending and 40% of per person federal inmate spending [68]. Thus, cost is a vital consideration for IHS, tribal governments, and tribally owned health systems when planning for implementation of health services in AI/AN communities.

We further situate this review within ongoing efforts to critique and strengthen the field of implementation science by elevating the insights and epistemologies of marginalized and under-represented communities, including those of Indigenous communities, and by rejecting oppressive or exclusionary forms of knowledge production [69, 70]. For example, noting that implementation science inadequately addresses systemic disparities designed to maintain racial inequalities, Bradley et al. draw on critical race theory and the Black radical tradition to help the field “center at the margins” to more effectively dismantle these systems of oppression that hinder access to health services [71]. Comparable reviews of implementation research applied to other types of health services in Indigenous communities have similarly noted that centering Indigenous epistemologies, using Indigenous research methodologies, building in extensive community participation, and paying attention to cultural safety will all help to mitigate epistemic injustice and improve the science [72, 73]. Such work has clear practical benefits: for example, the successful implementation of COVID-19 vaccination in Indigenous communities – with vaccine uptake rates in the US that were the highest among US race and ethnic groups – has been attributed to the centering of Indigenous practices and principles within those efforts [74, 75]. Even when applied to non-Indigenous or non-marginalized communities, implementation science would likely benefit from these epistemologies and practices. For example, implementation sustainability research could grow by integrating the Indigenous principles of Seventh Generation philosophy, or the idea that we should move through the world while keeping in mind the next seven generations of Earth’s inhabitants [76].

To maximize the potential benefit of future HIV implementation science for Indigenous communities, we argue that studies should be anchored to several guiding principles. First, respect for Indigenous sovereignty must be paramount. Interventions, implementation strategies, and implementation studies must be developed in meaningful partnership – recognizing and acknowledging the multiple forms of Indigenous knowing, being, and doing inherent within Indigenous communities. Such implementation work benefits from the science and wisdom held within Indigenous communities and has potential to expand intervention reach via cultural and contextual relevance. Second, while cultural adaptation of existing interventions is valuable, there is a need for the development and evaluation of interventions by, with, and for Indigenous communities. This challenges the prevailing 'top-down' paradigm in implementation science, which often presumes the desirability of interventions that have been evaluated elsewhere. Often, such ‘evidence-based’ interventions are tested under highly controlled (i.e., RCT) study designs in well-resourced academic settings, including mostly WEIRD (white, educated, industrialized, rich, democratic [77]) participant samples. Lack of attention to the differences in development versus implementation contexts may limit generalizability and contribute to implementation failure in marginalized communities [78]. The systematic failure of implementation in marginalized contexts contributes to the inverse-prevention law, in which those who most need evidence-based interventions are the least likely to receive them. Rather than an overreliance on adaptation, which can often take the form of changing only surface elements of interventions to fit Indigenous communities (i.e., “tagging a feather on it” [79]), a more equitable approach is to build interventions in contexts with the least, rather than the most, resources [80]. Using participatory approaches to develop and evaluate HIV preventative and treatment interventions in partnership with Indigenous communities has a dual promise of addressing the inverse-prevention law and expanding reach via cultural alignment and responsiveness. Third, given ongoing resource constraints and deep mistrust in many Indigenous communities of health systems and policymakers [81], we argue that future implementation studies must consider higher-level barriers to implementation, or what we might call the social determinants of implementation success [82]. We hypothesize that HIV implementation studies that strive to understand and counteract the effects of historical and inter-generational trauma, alongside the impacts of multiple intersecting systems of oppression on Indigenous communities, will expand the uptake and reach of HIV preventative and treatment programs. Fourthly, a strengths-based approach should be adopted, identifying and leveraging the unique resources, resilience, and implementation facilitators inherent in Indigenous communities. This shifts the narrative from one of deficit to one of empowerment [83]. Fifth, the use of Indigenous research methods and implementation strategies should be prioritized, ensuring that the research process itself is culturally congruent and respectful, and builds from effective practices of healing and doing that are already present within Indigenous communities. Sixth, there is an urgent need for implementation scientists to build capacity for implementation research within Indigenous communities. Lastly, HIV implementation studies must respect and reflect diversity both within and across Indigenous communities. These communities are not monolithic; all have distinct histories, epistemologies, and practices.

Several limitations to our review approach should be noted. First, our search was confined to PubMed, potentially excluding relevant studies indexed in other databases. Second, we restricted our search to Indigenous communities in the Americas and the Pacific, excluding research conducted with numerous Indigenous and colonized communities around the world. Third, though we highlighted the role of community engagement in each study, our review did not systematically assess the depth or quality of such engagement. Future work should be done to assess the quality and depth of academic-community partnerships to understand the processes of community engagement that are linked with improved implementation outcomes. Finally, given the dynamic nature of implementation science and the rapidly evolving landscape of HIV prevention and treatment, our exclusive use of peer-reviewed, published studies may mean we have missed recent developments and ongoing studies.

## Conclusions

Despite these limitations, our review offers a foundation upon which HIV implementation research in Indigenous communities can build. Future studies must expand the scope of this research, particularly to address high-priority HIV prevention and treatment services like PrEP and long-acting injectable treatment, to consider higher-level determinants of implementation success, and to rigorously assess later-stage implementation outcomes including cost and sustainability. They could identify culturally safe strategies for expanding access to and uptake of PrEP in Indigenous communities, explore the role of traditional healers and people with lived experience in these strategies, point to the most effective policy-level strategies for ensuring governments and health systems meet treaty obligations and respect Indigenous sovereignty, and identify the implementation strategies that are most congruent with community engagement and most effective at healing the effects of historical and intergenerational trauma. Our study also underscores the potential for an Indigenous implementation science that is culturally safe, community-based, and participatory. Evidence source matters – interventions and implementation strategies that are developed and evaluated by, with, and for Indigenous communities, and that are grounded in Indigenous ways of knowing, being, and doing, are likely to be more successful than those imported and adapted from other settings. We argue for a strengths-based approach that builds from the healing power of Indigenous traditions while acknowledging the realities of historical and intergenerational trauma, racism, oppression, the chronic and systemic under-funding of healthcare, and broken treaty obligations. Relational implementation strategies could leverage strong ties and social networks in Indigenous communities [84]. An Indigenous implementation science could enhance the acceptability, reach and effectiveness of critical HIV preventive and treatment services in Indigenous communities while also honoring their self-determination, knowledge, wisdom, and strength.

## Supplementary Information

Below is the link to the electronic supplementary material.

Supplementary file1 (DOCX 48 kb)

## Data Availability

Data are available upon request.
