# Supplementary material for: Implementation Science for HIV Prevention and Treatment in Indigenous Communities: a Systematic Review and Commentary
Source: Curr HIV/AIDS Rep. 2024 Aug 9;21(5):237–56. doi: 10.1007/s11904-024-00706-z (PMC11377631; doi:10.1007/s11904-024-00706-z)
Supplement: Supplementary file 1 — Supplementary file1 (DOCX 48 kb) [file 11904_2024_706_MOESM1_ESM.docx]

**Implementation Science and the Indigenous HIV/AIDS Response**

Search Protocol

**Specific Aims**

1. Review the implementation research literature for HIV preventive and treatment interventions in indigenous communities.

**Methods**

***Search Strategy***

PubMed:

HIV

and

("implementation science" or "implementation outcome" or "d & i" or "d&I” or "dissemination and implementation" or acceptability or acceptable or adoption or adopted or appropriateness or appropriate or feasibility or feasible or fidelity or cost or reach or penetration or sustainability or sustainment)

and

("Indigenous Peoples"[Mesh] OR Indigenous OR Aboriginal OR Torres Strait OR Inuit OR Maori OR “American Indian” OR “Native American” OR “Alaska Native” OR “First Nation” OR “Métis” OR “Native Hawaiian” OR “Pacific Islander” OR “Melanesian” OR “Micronesian” OR “Polynesian”)

*Search Results* (June 14 2023):

| **Database** | **Number of results** | **Notes/Exclusions** |
| --- | --- | --- |
| PubMed | 450 | None |

*Search Results* (June 16 2023):

| **Database** | **Number of results** | **Notes/Exclusions** |
| --- | --- | --- |
| PubMed | 478 | None |

*Search Results* (June 29 2023):

| **Database** | **Number of results** | **Notes/Exclusions** |
| --- | --- | --- |
| PubMed | 484 | None |

Other sources:

- Reference lists of included articles
- “Cited by” lists of included articles

***Inclusion/Exclusion Criteria*:**

**Study participants:** Any

**Intervention target population:** Indigenous communities from North, Central, and South America, Australia and Torres Strait Islands, New Zealand, and other Pacific Islands (Melanesia, Micronesia, and Polynesia)

**Intervention of interest**: Any HIV treatment or preventive intervention

**Outcomes**: Reporting or intending to measure at least one implementation outcome

**Setting:** North, Central, and South America, Australia and Torres Strait Islands, New Zealand, and Pacific Islands (Melania, Micronesia, and Polynesia)

**Types of studies:** We will include any study design, including study protocols. These will include but are not limited to: formative/qualitative studies (e.g., contextual determinants and mapping to implementation strategies), case studies, pilot evaluations, uncontrolled evaluations, quasi-experimental evaluations, RCTs, economic evaluations, policy analyses. Systematic reviews, meta-analyses, and commentaries are excluded.

**Additional limits, e.g. language, publication type:** No language limitations. Original research published in peer-reviewed journal.

***Study selection process*:**

Extract articles -

1. Search each database.
2. Note the number of results from each database.
3. Export the results to a common Covidence database and remove duplicates
4. Note the number of results with duplicates removed.

Review titles/abstracts of all articles -

1. Two reviewers screen every article in database for inclusion
   1. Screen title first
      1. If title indicates any potential for inclusion, read abstract
   2. Criteria for inclusion in the full-text review include: 1) reference to implementation of health-related intervention in title, abstract, or keywords; and 2) reference to LMIC in the title, abstract, and/or key words.
   3. If the reviewer is unsure about whether the article should be included in the full-text review, include a comment. These articles will be discussed by the group to decide on inclusion in the full-text review.
2. Reviewers meet regularly to discuss disagreements. If unable to come to consensus at title/abstract level, err on inclusion.
3. Note number of articles included after screening and number excluded.

Review of full text articles -

1. 2 reviewers independently download and read full text of articles included. Assess eligibility for inclusion.
   1. Studies must meet all criteria: 1) formative research and/or use of an ERIC implementation strategy, 2) in a LMIC setting
   2. For studies not meeting criteria above, note how they do not meet the criteria.
2. Note number of articles included.
3. Note number and reasons for articles excluded.

Data abstraction –

1. Develop data abstraction form which will include columns for author, date, setting, target health condition, target intervention, implementation strategy, implementation strategy specification, info on choice of implementation strategy and adaptation, study design, results, implementation outcomes, service delivery outcomes, health outcomes, other outcomes.
   1. Test the abstraction form with ~2 articles, revise the form, and then test with an additional ~2 articles.
2. Complete data extraction forms for articles to be included:
   1. One primary reviewer will be randomly assigned to each article, and they will abstract data for included article. One secondary reviewer will be randomly assigned to validate abstraction.

The data abstraction form will be reviewed for each article and discrepancies will be resolved by the group.

Systematic Review Flow Chart

Records excluded
(n =)

Records screened
(n =)

Records after duplicates removed
(n =)

## Identification

## Eligibility

## Included

## Screening

Records identified through database searching
(n =)

Full-text articles excluded, with reasons
(n =)

*Not target setting: n=*

*Not stigma reduction: n=*

*No IS outcomes: n=*

*>1 of above: n=*

Studies included in qualitative synthesis
(n =)

Full-text articles assessed for eligibility
(n =)

Additional records identified through other sources
(n = )
